# Social Support and Smoking during Pregnancy

**DOI:** 10.4172/2167-0420.1000179

**Published:** 2014-08-03

**Authors:** Saba W Masho, Elizabeth Do, Sulola Adekoya

**Affiliations:** 1 Department of Epidemiology and Community Health, Institute of Women’s Health and Center on Health Disparities, Virginia Commonwealth University, Richmond, USA; 2 Virginia Institute for Psychiatric and Behavioral Genetics, Virginia Commonwealth University, Richmond, USA; 3 Richmond Health District, Virginia Department of Health, Richmond, USA

**Keywords:** Prenatal smoking, Social support, Interpersonal support, Pregnancy, Tobacco, Smoking, Support, Perinatal health

## Abstract

**Background:**

Smoking during pregnancy and a lack of social support have been identified as independent risk factors for poor birth outcomes. However, the influence of social support on smoking during pregnancy remains under-investigated. This study examined the association between domains of social support and smoking during pregnancy.

**Methods:**

Pregnant women during their first trimester, attending three inner-city clinics were surveyed using self-administered questionnaires (N=227). Social support was measured using the Interpersonal Support Evaluation List (ISEL). Three domains of social support (tangible, appraisal, and belonging) were examined. Multiple logistic regressions were conducted; Odds Ratios (OR) and 95% confidence intervals were calculated.

**Results:**

Per unit increase in the total composite social support scale, there was a 6% increased odds of smoking during pregnancy. There was a statistically significant interaction between race and social support. While the tangible support (OR=1.15; 95% CI: 1.03, 1.27) and appraisal (OR=1.17; 95% CI: 1.05, 1.31) domains were significantly associated with smoking among African American women, only the belonging support domain was significantly associated with smoking during pregnancy among Caucasian women (OR=1.20; 95% CI: 1.02, 1.40).

**Conclusions:**

This study provided evidence that racial differences may exist in the way social support influences smoking during pregnancy. Future studies are needed to understand these racial differences and assist in the design of interventions. Considering the importance of social support, strategies for smoking cessation intervention should consider racial difference.

## Introduction

Adverse pregnancy outcomes are major problems in the United States. In an effort to address this problem, researchers continue to investigate risk and protective factors influencing these outcomes. Both smoking during pregnancy and a lack of social support have been identified as independent risk factors for adverse birth outcomes [1–3]. However, the relationship between these two factors in pregnant women remains understudied.

Smoking has been reported as one of the most harmful exposures during pregnancy [4]. It has been causally linked to poor birth outcomes; including Sudden Infant Death Syndrome (SIDS), low birth weight, and preterm delivery of the infant [5–7]. Women who smoke during pregnancy are 1.6–2.9 times more likely to have babies with low birth weights compared to non-smokers [8]. Moreover, infants of smokers are 150–250 grams lighter than those of non-smokers [9]. Smoking pregnant women are also 1.2–1.6 times more likely to have preterm births [10], and 1.3–1.8 times as likely to have stillbirths, when compared to non-smokers [11].

In general, disadvantaged women, including those who live in poverty, have low income and report low educational attainment are more likely to smoke during pregnancy [12–14]. Although the prevalence of smoking during pregnancy is higher among Non-Hispanic Whites, African Americans are disproportionately affected by poor pregnancy outcomes [15]. It has been postulated that social support can provide emotional and instrumental resources that in turn will impede the stressors on pregnant women, improving the outcomes of the pregnancy [16,17]. Considering the racial differences in poor pregnancy outcomes and smoking during pregnancy, it is important to examine the influence of social support on smoking and if the relationship differs by race.

Social support may be defined more broadly as the “process of interaction in relationships which improves coping, esteem, belonging, and competence through actual or perceived exchanges of physical or psychosocial resources” [18]. Social support can be classified into tangible, appraisal and belonging support. While the tangible support addresses perceived availability of material aid, the appraisal and belonging subdomains focus on emotional support [19]. Considering smoking is a substance used in a social setting and associated with stress, the different types of social support may influence smoking during pregnancy differently. Much of the literature thus far, has been focused on the influence of social support as it relates to stress [20–22]. Further, higher level of stress is associated with smoking during pregnancy, relapse and difficulty in smoking cessation [23]. However, the impact of social support during pregnancy on poor lifestyle behaviors such as smoking has not been well understood. The current body of literature is unable to demonstrate a consistent association between smoking during pregnancy and social support. Whereas some researchers have found little to no relationship between social support and tobacco use during pregnancy, others have found increased or decreased substance use among women with low levels of social support [24–31]. Both the magnitude and direction of this association remain unclear. Further, the sub domains of social support as they relate to smoking during pregnancy are not investigated. Considering the persistent racial disparities in poor pregnancy outcomes, racial differences in the relationship between social support and smoking during pregnancy are under studied.

Thus, this study seeks to investigate the association between social support and smoking during pregnancy as related to the domains of social support, including, tangible, appraisal, and belonging support. Additionally, the study will examine racial difference in the relationship between social support and smoking.

## Methods

Pregnant women (N=227) in their first trimester attending prenatal care were surveyed from February 2010–July 2012 using a self-administered questionnaire. Study participants were recruited from a major university hospital, health department and private clinic. The clinics predominantly serve low income pregnant women. Study participants were 18 years or older, English speaking and were able to consent. Survey questionnaires were administered using paper and pencil at the clinics. Current smoking status was assessed in the questionnaire using the following question: “How many cigarettes do you smoke on an average day now? (A pack has 20 cigarettes)”. The responses to this question were “41 cigarettes or more”, “21 to 40 cigarettes”, “11 to 20 cigarettes”, “6 to 10 cigarettes”, “1 to 5 cigarettes”, “none (0 cigarettes)”. These responses were categorized into “current smoker,” indicating the respondent had more than 1-cigarettes and “non-smoker”, indicating that the respondent had none (0 cigarettes).

Social support was measured using the short version of the Interpersonal Support Evaluation List (ISEL) [32]. The ISEL was designed to assess the perceived availability of three separate functions of social support as well as provide an overall support measure. The domains that comprise social support are: a) “tangible” subscale, which measures the perceived availability of material aid; b) “appraisal” subscale measuring the perceived availability of someone to talk to about one’s problems; and c) “belonging” subscale, the perceived availability of people one can do things with. This scale is validated and has been widely used to measure social support [19]. We also created a composite ISEL score indicative of total social support by combining the three domains.

Sociodemographic factors such as age, race, ethnicity, marital status, education, and income were examined. Only 12 women identified themselves as Hispanic; all of these Hispanic women identified their race as Caucasian and none reported smoking. As a result, we were not able to assess ethnicity in the context of this study. However, these women were retained in the data as Caucasians. In addition to demographic variables, reproductive history including: number of pregnancies, number of children, previous preterm births, and pregnancy intentions, were assessed. The Adequacy of Prenatal Care Utilization Index (APNCU)was calculated to evaluate the adequacy of prenatal care. Women were also asked if they were currently using alcohol or illicit substances such as cocaine, marijuana, and heroin. Furthermore, current stress level was assessed using the perceived stressed scale [33]. Exposure to stressful events was also assessed using a modified version of the stressful life events inventory (SLEI) which evaluated lifetime exposure as well as past year exposure [34].

A composite score was created from each domains of the ISEL and analyzed as a continuous variable. All three domains of the ISEL and the composite score were examined as continuous variables and analyzed in association with smoking status. Descriptive findings were reported using frequencies, means and percentages. Because the interrelationship between race, social support and smoking during pregnancy has not been well understood, race was tested as a potential effect modifier. A test for interaction using univariate regression analysis showed that race was a statistically significant effect modifier in the association between social support and smoking and data was stratified by race. Multivariable analysis was conducted using logistic regressions, and odd ratios (OR) and 95% Confidence Intervals (CI) were calculated. Potential confounders were identified according to previous literature and retained in the model, if each results in 10% change in the estimate. The model was built from the univariate analysis by adding each confounder and retaining those that resulted in 10% change in estimate. Additionally, the full model that has all the variables was assessed and variables were dropped. The model with marital status and education was found to be the most parsimonious model. Adjusted models for tangible, appraisal, and belonging support domains and the total composite social support were assessed independently. This study was approved by the Virginia Commonwealth University Institutional Board.

## Results

The average age of the study participants was 25.7 years. A majority of the women were Black/African American (68.7%), not married (82.7%), had high school or less education (57.3%), employed (60.2%) and earned less than $20,000 (74.5%). Over a quarter of the women were smokers (26.4%) (Table 1). The unadjusted analysis showed that there was no statistical difference between smokers and non-smokers in terms of age, race, insurance status, alcohol use, adequacy of prenatal care, intimate partner violence, and intention of the pregnancy (Table 2). However, being unmarried, having lower levels of education, annual household income under $20,000, being unemployed, having experienced previous preterm birth, and illicit drug use were significantly associated with smoking during pregnancy. Marital status and education level were found to be statistically significant confounding factors.

The adjusted analyses showed that there was a significant association between social support and smoking (Table 3). Per unit increase in the total composite social support scale, there was a 6% increased odds of smoking during pregnancy (OR=1.06, 95% CI=1.02, 1.10) for all women (prior to stratification). This association was significant for African American women (OR=1.06; 95% CI: 1.01, 1.11), but not for Caucasian women. The tangible support domain was significantly associated with smoking for all participants (OR=1.06; 95% CI: 1.03, 1.22) and for only African American women when stratified (OR=1.14; 95% CI: 1.03, 1.27). Appraisal support was significant for all study participants and African American women, but not for Caucasian women. For all study participants, per unit increase in the appraisal support domain, there were a 17% increased odds of smoking during pregnancy (OR=1.17; 95% CI: 1.06, 1.29). Smoking and the belonging support domain were significantly associated in Caucasian women but not in African American women. Specifically, Caucasian women with higher levels of belonging social support had greater odds of smoking during the first trimester of pregnancy (OR=1.20; 95% CI: 1.02, 1.40).

## Discussion

In this study, increased social support was found to be significantly associated with smoking in the first trimester of pregnancy. The study also reported racial differences in the association between the different types of social support and smoking during pregnancy. While tangible and appraisal support were significantly associated with smoking among African Americans, belonging support was the only domain significantly associated with smoking in Caucasians. Few studies have reported a strong relationship between social support and smoking during pregnancy and no previous studies, to the knowledge of the authors, have investigated racial differences. A recent study by Elsenbruch, et al. reported that women with low social support were more likely to smoke during the first trimester of pregnancy compared to women with high social support (i.e. 34% Vs. 17%, respectively) [36]. However, this study was conducted in Germany where the population is more homogeneous and different from the population in this study. Another study conducted in 2008 by Cannella found that increased social support was associated with positive health practices, including smoking cessation [17]. However, this study used a convenience sample that was largely homogenous and the findings may not be comparable to our study. By and large, existing literature has provided inconsistent findings [17,19,25–30,32,35–38]. While some studies reported that social support was positively associated with smoking during pregnancy, others have shown a weak association or a negative association. This inconsistency in the literature may be due to differences in population, methodology and confounding factors and interaction terms examined.

The findings of this study reported a positive association between social support and smoking during the first trimester of pregnancy. Compared to other studies [36], this study reported a modest association between social support and smoking during the first trimester of pregnancy. Although this study was a cross-sectional study and was not able to assess causality, it provided the evidence that women who smoked during the first trimester of the pregnancy also had strong social support. The social support observed among smokers could potentially be a conduit for intervention. Pregnancy is a time when women share their experiences and seek or elicit support from their network if distressed [39–42]. It is important to recognize that their support system may provide an opportunity for intervention.

This study reported that there were important racial differences in the association between the types of social support and smoking during the first trimester of pregnancy. Among African American/black women, smoking during the first trimester was associated with tangible and appraisal support; while belonging support is associated with smoking among Caucasian women. The association with tangible support in African Americans/blacks but not in Caucasians indicates the importance of material support in African Americans/blacks. Material distress can be stressful and is known to be associated with smoking during pregnancy [16,17]. The belongingness domain had the strongest influence on smoking among Caucasian women. The belongingness domain indicates the perceived availability of people one can do things with and share experiences. This finding suggests that social network or family and friends may have stronger influence in the smoking behavior for Caucasian pregnant women.

Moreover, understanding the influence of the different types of social support by race is very useful in creating smoking cessation interventions during the preconception or in the earlier stages of pregnancy. While programs to reduce material stressors could be targeting women with a need for tangible support, social networks could be targeted for belonging support. For instance, a study by Koshy et al., 2010, reported that women who quit smoking during pregnancy claimed receiving higher amounts of active praise and encouragement than those who did not quit smoking [31]. This suggests that the importance of understanding the type of social support to effectively target women who can benefit from the intervention. A recent qualitative study conducted by Nguyen et al. reported that women who decided to quit smoking during pregnancy were often enmeshed in social networks with prominent smoking norms, tempted to smoke by members of their social networks, and experienced changes in their relationships with smokers within their social network upon the decision to stop smoking. These changes entailed: alteration in how they felt about how smokers perceived them, loss of connection, or isolation [41]. Thus, in order to preserve their social networks and obtain the social support they need during pregnancy, women may choose to continue smoking, despite the risks that it may pose to their offspring. Since these changes seem to be reflective of changes in belonging, appraisal or tangible support or the perception that there is a group with which one can identify and socialize, future interventions focused on maternal smoking cessation should look towards incorporating social networks into their conceptual framework [41]. This is especially important for intervention targeting different racial groups during the preconception or first trimester of pregnancy.

The reason for the association in the belonging support domain among Caucasian American but not African American pregnant women is unknown. It is also unclear why tangible support, appraisal support, and total social support was significantly associated for African American pregnant women but not among Caucasian women. However, it is possible that social networking plays a very important role in Caucasians and addressing material needs may be more important for African Americans/blacks. Considering race is a social construct, it is possible that social networking or the influence of social support is different in these two populations. Clearly, further research will be needed to assess if this relationship is causal. However, this study has provided the evidence that the type of social support differ by race.

The finding of this study has some important public health implications. The study provided insight to understand the role of social support that may be important in designing interventions to prevent maternal smoking in the earlier stages of pregnancy. Considering the role of belonging support in Caucasians, it may be important for programs using individualized or group-based interventions to consider social supports. Individualized interventions may be focused on providing one-on-one counseling to discuss strategies and also getting women to call quit lines or provide them with other resources. However, it is important that these intervention programs actively involve the woman’s partner or other influential members of her social network [43]. A recent study conducted in 2010 by Hennrikus et al. identified pregnant smokers as well as a woman in their social network to help them quit smoking and the dyads were randomized into intervention and control groups. Women reported that their female friends and family supports were helpful in adopting healthy behaviors. The quit rates in the intervention group (13%) were significantly higher compared to the control group (3.6%), demonstrating that cessation rates were influenced by increased frequency and quality of support through a woman’s social network [43]. Further, the study showed that pregnant women who received support from friends were more likely to quit than those supported by family members (21.7% vs. 6.5%).

The finding that tangible support is associated with smoking among African Americans/blacks has significant practical implication. The lack of material resources can be a major stressor during pregnancy and stress is known to be associated with smoking during pregnancy [16,17]. This information is helpful for case management programs that provide significant support to pregnant women. Additionally, understanding the role of social support may be beneficial to prenatal home visitation programs, as they have reported success in decreasing maternal smoking while also providing social support, community services and resources, and intensive counseling for other negative health behaviors [44].

The finding of this study may be relevant to public health professionals and providers who work with group based interventions. A study investigating network phenomena on smoking cessation suggested that decisions to quit smoking are not made by sole individuals, but rather is a reflection of a choice made by groups of people within a network. Thus, changes in smoking behaviors of one or more people within a social network may be required for a pregnant woman to quit [45]. In the context of women who smoke during pregnancy, it may be useful to provide smoking cessation programs in tandem with prenatal care in group-settings like in the Centering Pregnancy approach, described elsewhere [46]. This would provide pregnant women with a social network that will support them in smoking cessation that they might not receive in their other social networks. These intervention strategies can be shaped by formative research. Specifically, longitudinal studies can be conducted to aid researchers in assessing substance abuse fluctuations during pregnancy [42].

This study was able to examine the role of social support on a very important preventable risk factor, smoking. The ability of the study to examine each of the domains by race is an important strength of this research. However, this study has a number of limitations. First, the study was unable to examine smoking status throughout the pregnancy and did not examine smoking cessation. The finding of the study is limited to smoking in the first trimester of pregnancy and does not provide information on smoking or social support later in pregnancy. Second, this study employed a convenience sampling design and findings from this study cannot be generalizable to populations other than the study population. In addition, the majority of the participants were non-Hispanic African American women, which limits the generalizability of these findings to other populations. However, the study shades light on the potential relationship between social support and smoking during pregnancy. Third, smoking is self-reported data and it is likely that some smokers may have denied their smoking status and been misclassified as non-smokers. This may have resulted in under estimation of the association between social support and smoking. Fourth, due to the cross-sectional nature of the study design, this study is unable to show temporality or causal relationship. However, the finding of the study provides evidence that there is an association between social support and smoking in the first trimester of pregnancy. Lastly, only marital status and education were found to be significant confounders. It is possible that other covariates were not significant due to the small sample size employed in this study.

In conclusion, this study reported a statistically significant association between social support and smoking among pregnant women and indicated that this association may vary as a function of racial differences. Future studies are needed to understand these racial differences and assist in the design of interventions. Longitudinal studies with larger sample size are needed to fully examine the relationship and account for additional factors that may influence this association.

## Figures and Tables

**Table 1:** Characteristics of Women Attending Three Prenatal Care Clinics between 2010–2012 in Richmond, Virginia.

Characteristics	Total (%) N=227	Black/African American N=156	White/Other N=71	Chi-Square t-test	P-value
**Mean age in years (SD)**	25.7 (5.0)	25.0 (4.7)	27.7(5.7)	2.43	0.45
**Marital Status** **Married** **Not married**	18.6 81.4	5.5 94.5	46.7 53.3	45.76	<0.0001
**Highest level of education** **<High school** **High school or GED** **Some college or greater**	22.5 34.8 42.7	25.0 42.4 32.6	11.1 19.1 69.8	23.97	<0.0001
**Annual household income** **<$20,000** **≥ $20,000**	74.5 25.5	86.2 13.8	46.8 53.2	31.49	<0.0001
**Employment status** **Unemployed**	39.8	46.0	24.2	8.34	0.004
**Insurance** **Not Insured**	60.6	67.4	45.2	8.73	0.003
**Parity** **None** **One** **Two or more**	49.8 26.0 24.2	53.8 21.2 25.0	46.0 33.3 20.7	3.34	0.19
**Pregnancy Intention** **Yes**	25.8	16.9	39.7	11.93	0.0001
**Previous Preterm** **Yes**	14.2	14.4	13.3	0.35	0.55
**Alcohol Use** **Yes**	59.0	60.7	68.3	0.75	0.39
**Illicit drug use**^a^ **Yes**	13.2	16.67	9.5	1.77	0.18
**Physical Abuse** **Yes**	5.8	6.1	7.9	0.21	0.64
**Stressful Life event Inventory**^b^ **Low** **Medium** **High**	35.1 34.1 30.8	36.4 32.6 31.0	38.1 27.0 34.9	0.66	0.72
**Social Support**^c^ **Low** **Medium** **High**	32.2 32.2 35.7	31.1 37.8 31.1	36.5 28.6 34.9	1.64	0.44
**Kotelcheck Index** **Inadequate/Intermediate** **Adequate** **Adequate Plus**	15.9 79.0 6.1	15.9 80.3 5.9	15.9 76.2 6.0	3.81	0.43
**Smoking**	26.4	28.9	21.1	1.49	0.22

a Illicit drug use include cocaine, Marijuana, heroin, or methamphetamines

b stressful life events inventory (SLEI)

c Interpersonal Support Evaluation List (ISEL)

**Table 2:** Factors Associated with Smoking during First Trimester of Pregnancy: Unadjusted Analysis.

Characteristics	OR (95% Confidence Interval)		
	Total	Black	White/other
**Maternal age in years**	1.01 (0.95–1.07)	1.09 (1.01–1.18)*	0.86 (0.75–0.98)*
**Not married**	3.61 (1.22–10.68)*	§	2.86 (0.81–10.12)
**Highest level of education**			
**< High school**	4.18 (1.95–8.94)***	3.06 (1.24–7.59)**	6.83 (1.51–30.83)
**High school or GED**	1.49 (0.72–3.11)	1.06 (0.44–2.57)	2.73 (0.65–11.56)
**Some college or greater**	Reference (1.00)	Reference (1.00)	Reference (1.00)
**Income <$20,000**	3.84 (1.54–9.57)**	3.70 (0.81–16.98)	3.83 (1.08–13.54)*
**Unemployed**	2.62 (1.40–4.88)**	2.75 (1.30–5.80)**	2.10 (0.63–7.03)
**Not Insured**	1.17 (0.64–2.15)	1.11 (0.53–2.33)	1.05 (0.33, 3.30)
**Unintended pregnancy**	1.55 (0.75–3.17)	1.84 (0.70–4.85)	0.97 (0.30–3.11)
**Parity**			
**None**	Reference (1.00)	Reference (1.00)	Reference (1.00)
**1**	1.26 (0.60–2.65)	1.69 (0.69–4.13)	0.69 (0.18–2.66)
**2+**	2.29 (1.13–4.64)*	3.43 (1.49–7.90)*	0.72 (0.16–3.19)
**Previous preterm birth**	3.49 (1.61–7.55)**	2.75 (1.11–6.82)*	6.38 (1.45–27.98)*
**Kotelchuck Index**			
**Inadequate/Intermediate PNC**	0.86 (0.39–1.92)	0.99 (0.40–2.42)	0.52 (0.08–3.26)
**Adequate PNC**	Reference (1.00)	Reference (1.00)	Reference (1.00)
**Adequate Plus PNC**	1.08 (0.43–2.71)	0.96 (0.34–2.72)	1.73 (0.22–13.67)
**Alcohol Use**	0.73 (0.40–1.32)	0.99 (0.49–2.00)	0.30 (0.09–1.00)*
**Illicit drugs**	3.97 (1.80–8.77)***	4.56 (1.84–11.28)**	2.00 (0.33–12.13)
**Intimate partner violence**	2.51 (0.81–7.79)	1.49 (0.34–6.54)	6.75 (1.01–44.90)*
**Mean Number of Stressful Life Events**			
**Lifetime**	1.07 (1.02–1.12)**	1.04 (0.99–1.10)	1.19 (1.07–1.33)**
**Year**	1.13 (1.05–1.21)***	1.11 (1.02–1.20)*	1.16 (1.03–1.31)*
**Social Support**			
**Tangible support**	1.12 (1.03–1.21)**	1.12 (1.02–1.23)*	1.11 (0.97–1.27)
**Appraisal support**	1.20 (1.09–1.32)***	1.18 (1.06–1.32)**	1.24 (1.01–1.51)*
**Belonging support**	1.06 (0.98–1.15)	1.00 (0.91–1.11)	1.21 (1.04–1.40)*
**Total Composite score**	1.06 (1.02–1.10)**	1.05 (1.00–1.09)*	1.08 (1.01–1.16)*

§ The number of African Americans who are married were very small and OR couldn’t be calculated.

* p-value < 0.05

** p-value <0.01

*** p-value <0.001

**Table 3:** Association between Social Support and Smoking by Race: Adjusted Analysis.

Social Support	OR (95% CI)		
	Total	Black	White/Other
**Tangible Support**	1.12 (1.03–1.22)**	1.15 (1.03–1.27)*	1.09 (0.93–1.27)
**Appraisal Support**	1.17 (1.06–1.29)**	1.17 (1.05–1.31)**	1.17 (0.94–1.44)
**Belonging Support**	1.08 (0.99–1.17)	1.04 (0.93–1.15)	1.20 (1.02–1.40)*
**Total Support**	1.06 (1.02–1.10)**	1.06 (1.01–1.11)*	1.07 (1.00–1.15)

All models adjusted for marital status and education

* p-value < 0.05

** p-value <0.01
